# Nurse night shift work and risk of gastrointestinal cancers

**DOI:** 10.3389/fpubh.2025.1532623

**Published:** 2025-04-28

**Authors:** Lin Guo, Xiaojun Li

**Affiliations:** ^1^Medical Simulated Center, Inner Mongolia Medical University, Hohhot, Inner Mongolia, China; ^2^School of Nursing, Inner Mongolia Medical University, Hohhot, Inner Mongolia, China

**Keywords:** circadian, night shift, gastrointestinal, cancer, colon cancer, colorectal cancer

## Abstract

The prevalence of night-shift employment is on the rise among full-time and part-time workers globally. Those engaged in night-shift work encounter various biological challenges, including exposure to artificial light during nighttime and disruptions to their circadian rhythms. These factors, along with changes in daily routines and activities, may pose significant risks to the health of night workers. Notably, the number of individuals working overtime or on night shifts has increased across various sectors, particularly in transportation, healthcare, and manufacturing. The International Agency for Research on Cancer (IARC) has classified night-shift work as probably carcinogenic to humans (IARC Group 2A). Subsequent research has identified several potential mechanisms through which night-shift work may contribute to carcinogenicity: (1) disruption of circadian rhythms, (2) suppression of melatonin levels due to nighttime light exposure, (3) physiological alterations, (4) lifestyle changes, and (5) reduced vitamin D levels resulting from inadequate sunlight exposure. Colorectal cancer (CRC) poses a significant public health challenge, ranking as the second leading cause of cancer-related death worldwide in 2020. Other than CRC, other gastrointestinal cancers are also creating a great global health issue because of their morbidity and mortality rates. In this review, we highlight the role of night shifts in disturbing circadian rhythm and how this action leads to carcinogenesis in the GI tract.

## 1 Gastrointestinal cancers

### 1.1 Epidemiology

Colorectal cancer poses a significant public health challenge, ranking as the second leading cause of cancer-related death worldwide in 2020, according to GLOBOCAN estimates from the International Agency for Research on Cancer (IARC) (1). This analysis reveals a stark geographic disparity in CRC mortality, with Europe exhibiting the highest age-standardized mortality rate (ASMR) at 12.3 per 100,000 person-years, while Africa and the Eastern Mediterranean region (EMRO) report the lowest rates at 5.6 and 5.3, respectively. Furthermore, a notable gender a disparity exists, with men experiencing a significantly higher ASMR (11.0) compared to women (7.2) (1). CRC also emerged as the third most prevalent cancer globally in 2020, with an estimated 1,931,590 new cases reported. Similar to mortality trends, geographic variations in incidence rates were observed, with Europe exhibiting the highest age-standardized incidence rate (ASR) at 30.4, while EMRO and Africa displayed the lowest rates at 9.1 and 8.4, respectively. Strikingly, high-income countries demonstrated a nearly four-fold higher ASR (30.2) compared to low-income (8.8) and low-middle-income (7.4) countries, highlighting the potential influence of socio-economic factors on CRC incidence (2). Analysis of temporal trends reveals contrasting patterns in CRC incidence across the globe (3–5). While developed countries previously classified as high-risk have observed stable or even slightly declining trends in recent decades, countries with historically lower CRC risk, such as Brazil, Costa Rica, and India, have experienced notable increases in incidence (6). These shifts in incidence patterns, coupled with the projected substantial increase in new CRC cases globally, warrant further investigation into the underlying risk factors and potential interventions to mitigate the burden of this disease (7–9).

### 1.2 Etiology

Hereditary mutations are only attributed to only approximately 20% of CRC cases, which are called sporadic CRCs. This suggests that environmental factors such as lifestyle, dietary habits, and the composition of the microbial community play a significant role in the initiation of tumorigenesis. Sporadic CRC typically arises following mutations in the adenomatous polyposis coli (APC) gene, which sets off a series of events culminating in CRC development (10).

APC serves as a negative regulator of the WNT/*β*-Catenin signaling pathway, which when upregulated, is linked to cancer progression. Further mutations in additional tumor suppressor genes, including tumor protein 53 (TP53) and Kirsten rat sarcoma viral oncogene homolog (KRAS), facilitate the process of malignant transformation (11). There is an association between near 15% of CRC cases and heritable mutations or epigenetic silencing of mismatch repair genes, leading to mismatch repair deficiency (MMRd) and an increased prevalence of mutations in repetitive DNA sequences known as microsatellite instability (MSI-high), thereby heightening the risk of mutations driving CRC (12). Additionally, chronic inflammation may induce dysplastic changes, resulting in colitis-associated cancer (CAC) (13). Although similar driver mutations are involved in CAC, the occurrence and timing of these mutations differ, with TP53 mutations commonly arising before those in APC. Regardless of the underlying cause, most CRC cells exhibit activation of pathways driving survival, proliferation, or immune response, including WNT/*β*-Catenin, nuclear factor-κB; NF-κB, and signal transducer and activator of transcription 3 (STAT3) (14–16).

### 1.3 Diagnosis

The preliminary assessment for colon cancer may include a barium enema or CT colonography. However, a definitive diagnosis necessitates a colonoscopy to obtain tissue samples (17–19). The sensitivity of colonoscopy can reach approximately 94.7% when conducted by a skilled practitioner with proper bowel preparation. It is important to note that 2 to 6% of lesions, particularly those that are right-sided, sessile, or flat, may be overlooked during the procedure. A comprehensive colonoscopy should encompass multiple biopsies of any suspicious lesions. In addition, the surrounding colon tissue adjacent to the tumor area should be marked to aid in intraoperative localization. Standard laboratory evaluations should consist of a complete blood count, iron studies, basic metabolic panel, liver function tests, and coagulation assessments, as chronic tumor-related bleeding can result in iron deficiency anemia. Carcinoembryonic antigen (CEA) is the predominant tumor marker utilized in colon cancer and should be established at baseline. Elevated CEA levels are associated with a worse prognosis, and post-treatment CEA measurements are valuable for monitoring potential disease recurrence. The National Comprehensive Cancer Network (NCCN) advocates for universal testing of MMR/MSI status in all patients diagnosed with colon cancer, given that this condition affects approximately 15 to 20% of sporadic colorectal cancer cases. Additionally, mutation testing for KRAS, NRAS, HER2, and BRAF is suggested for patients with non-respectable metastatic colon cancer. It is also recommended that on all individuals diagnosed with colon cancer, CT scans of chest, abdominal, and pelvic should be performed. Magnetic resonance imaging (MRI) is generally reserved for cases where liver metastases are suspected or when the patient has a known allergy to iodinated contrast agents. Routine use of positron emission tomography-CT scans is not advised (20).

### 1.4 Night shift work and its impact on gastrointestinal cancer risk

The growing body of research on night shift work and gastrointestinal cancer risk highlights the significant impact of circadian rhythm disruption on carcinogenesis. Shift workers, particularly nurses, experience misalignment in their biological clocks due to chronic exposure to artificial light at night, altered sleep patterns, and irregular meal timing. These disruptions influence various physiological processes, including hormone regulation, immune function, and metabolic pathways, all of which contribute to increased cancer susceptibility (21, 22). Studies have suggested that prolonged night shift work may be associated with an increased risk of colorectal cancer, particularly in individuals with over 15 years of exposure to rotating night shifts (23, 24). Disruptions in melatonin production, a key regulator of circadian rhythms with known anti-cancer properties, have been implicated in colorectal tumorigenesis (25, 26). Furthermore, night shift work has been linked to metabolic disturbances such as obesity and insulin resistance, both of which are established risk factors for gastrointestinal cancers (27–29). Despite these associations, findings remain inconsistent, and additional large-scale, nurse-specific studies are needed to determine the precise risk levels for different gastrointestinal cancers (30, 31). Future research should focus on identifying potential intervention strategies, such as optimizing shift schedules, improving sleep hygiene, and assessing the role of melatonin supplementation in mitigating cancer risk among night shift workers (26, 32).

## 2 Circadian rhythm: from physiology to pathology

### 2.1 Definition

The coordination of an organism with its external and internal environments is essential for its health and survival; if there is a mismatch between the organism and its surroundings, it could result in its annihilation. Circadian rhythms are controlled by an internal timing mechanism that operates at the transcriptional level, leading to gene networks that fluctuate in a 24-h cycle. These networks include clock genes that regulate the rhythms of physiological processes and behaviors (21, 22). The term circadian rhythm is derived from “circa diem,” or “about a day” and is used for defining the mechanisms by which align energy collection and usage activities with the sun’s movements during sunrise and sunset in all living systems. Initial studies on mammalian rhythms focused on rhythmic behaviors as indicators of the biological clock, identifying the hypothalamic suprachiasmatic nucleus (SCN) as the primary circadian pacemaker influencing these behaviors (21). SCN neurons can produce their own circadian rhythms. However, they have several unique features:

1. They receive direct light signals from the retina, enabling them to align with the day/night cycle.
2. They possess specialized and organized coupling mechanisms that help them maintain synchronization with each other even in the absence of light.
3. They create a distinct circadian rhythm in their neuronal firing frequency, enabling them to synchronize other cells in the body through various direct and indirect pathways.

SCN aligns itself with the light/dark cycle, enabling it to synchronize other smaller cellular oscillators. Additionally, due to its internal connections, the SCN produces a consistent output signal even without a light/dark cycle, which explains the “free-running” circadian rhythms (approximately 24 h) of physiological and behavioral processes that continue under stable conditions (33–37). However, the identification of ‘clock genes’ revealed that the ability for circadian gene expression is common throughout the body. These genes are observed to be different in distinct species. The importance of protein synthesis in the mammalian circadian pacemaker was confirmed in the late 1980s, and research into the basic molecular genetic oscillatory mechanisms commenced earnestly around a decade later. The initial mammalian circadian clock gene, Clock, was discovered through a forward-genetics mutagenesis screening, and this finding was soon followed by the identification of various other essential molecular clock components, some of which were similar to those found previously (21, 38).

### 2.2 Circadian rhythm at molecular levels

In mammals, the circadian clock functions through a self-sustaining mechanism that operates independently at the cellular level, stemming from a negative feedback transcriptional network that regulates itself. The central components of this clock in mammalian cells are the transcriptional activators CLOCK (and its closely related counterpart, NPAS2) alongside BMAL1. In the late 1980s, it was recognized that protein synthesis plays an essential role in the circadian pacemaker of mammals. Approximately ten years later, researchers began to thoroughly unravel the basic molecular genetic mechanisms behind these rhythmic patterns. The first circadian clock gene in mammals, known as Clock, was discovered through a forward-genetics mutagenesis study (38–40). These activators play a crucial role in enhancing the expression of the Period (Per1, Per2) and Cryptochrome (Cry1, Cry2) genes at the start of the cycle (41). The products of the Per and Cry genes build up, pair up, and create a complex that moves into the nucleus to engage with CLOCK and BMAL1, thereby inhibiting their own transcription. This feedback cycle lasts approximately 24 h, and the regulation of PER and CRY protein levels is meticulously controlled by E3 ubiquitin ligase complexes. Furthermore, there are other feedback loops connected to the main CLOCK-BMAL1/PER-CRY loop. A significant loop in this system involves Rev-erbα (Nr1d1) and Rora, which are direct targets of CLOCK-BMAL1. The feedback effects from this loop impact the transcription of Bmal1 (and to a lesser extent CLOCK), leading to an antiphase oscillation of BMAL1. The Nr1d1/2 genes produce the nuclear receptors REV-ERBα and REV-ERBβ. These receptors play a key role in cyclically inhibiting the transcription of the Bmal1 and Nfil3 genes, which are activated by the retinoic acid-related orphan receptors *α* and *β* (RORα/β). NFIL3, in combination with D-box binding protein (DBP), alongside CLOCK and BMAL1, modulates the rhythm of the REV-ERBα/β nuclear receptors. Additionally, another feedback loop includes the PAR-bZip family members, DBP, HLF, and TEF; the bZip protein E4BP4 (Nfil3); as well as the bHLH proteins DEC1 and DEC2 (Bhlhb2, Bhlhb3), all of which are also transcriptional targets of CLOCK-BMAL1. The particular roles of these different genes in the interconnected molecular feedback loops that produce circadian signals at the cellular level resulting in rhythms across a variety of physiological systems, including sleep, metabolism, and aging (41–43).

### 2.3 Retinohypothalamic tract

**Figure 1:**
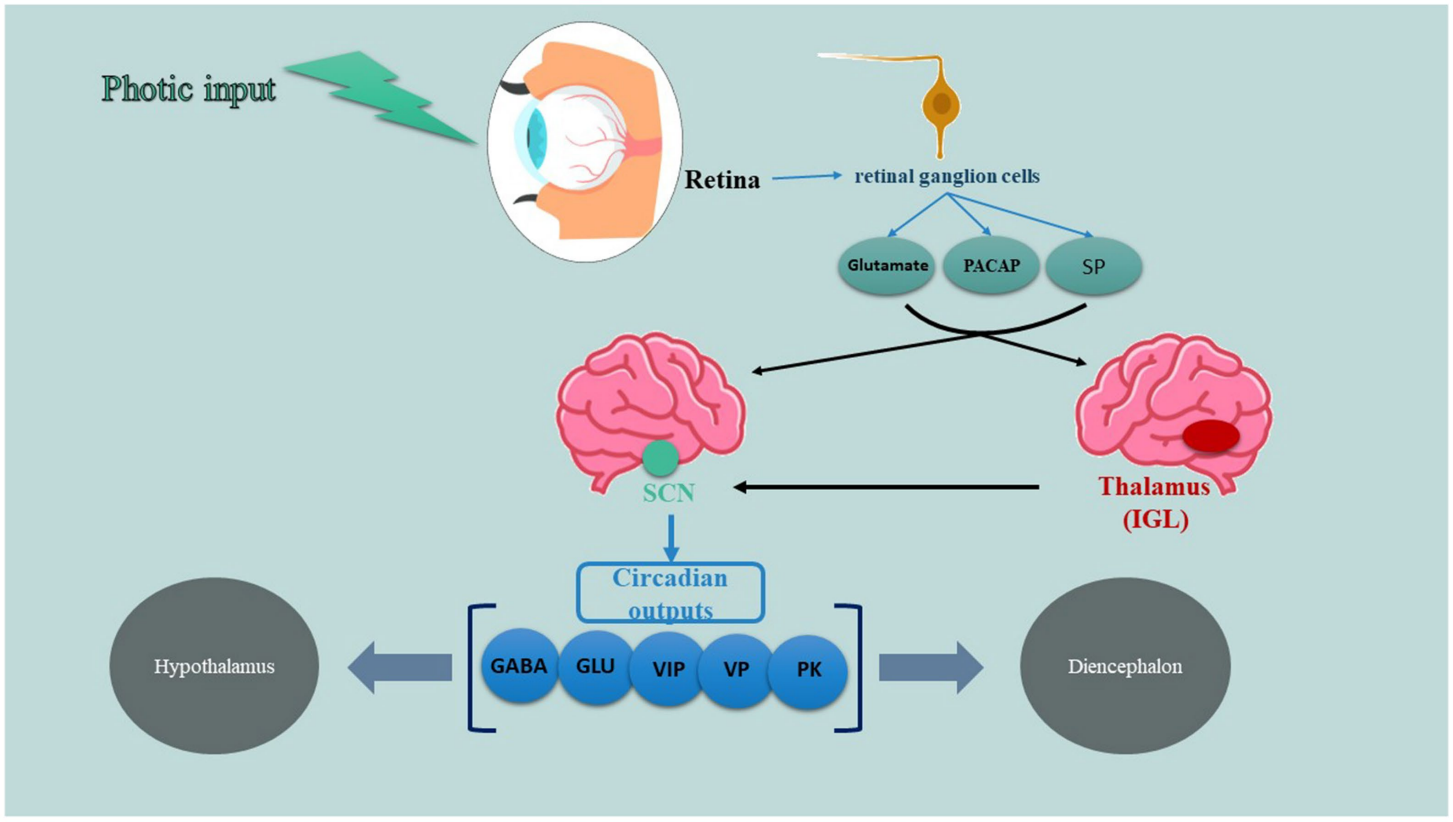
A specific system for retinal projection.

A specific system for retinal projection, known as the retinohypothalamic tract (RHT), is essential and adequate for the light-based regulation of the circadian pacemaker (also shown in Figure 1). The RHT comes from a unique group of retinal ganglion cells that are different from those responsible for the main visual pathways. It primarily connects to the SCN and has more minor connections to the anterolateral hypothalamus, subparaventricular zone, and supraoptic area. As well, the thalamic intergeniculate leaflet (IGL) is another key player of this system which RHT axon collaterals also project to (44, 45). According to studies, not only classic photoreceptors including rods and cons are involved in this tract but also photo-sensitive retinal ganglion cells (which use peptide melanopsin as a photopigment) are taking part in transducing signals to SCN. RHT terminals release glutamate, an excitatory amino acid neurotransmitter, which functions through N-methyl-D-aspartate (NMDA) receptors, non-NMDA receptors, and several intracellular signaling pathways to enhance the expression of the Per gene. These alterations in gene expression, occurring alongside the active circadian transcription-translation cycle, functionally correspond to phase shifts in the circadian oscillator. Besides glutamate, RHT terminals also discharge two recognized peptide cotransmitters: substance P (SP) and pituitary adenyl cyclase–activating peptide (PACAP) (46). SP appears to be crucial for RHT transmission, as specific SP antagonists inhibit light-induced phase shifts and immediate-early gene expression in living organisms, as well as glutamate receptor-mediated phase shifts in laboratory settings. Additionally, the administration of SP can trigger circadian phase shifts on its own. In contrast, the administration of PACAP has been noted to either counteract or replicate the effects of glutamate on circadian phase shifting and Per gene expression in laboratory settings, depending on the dosage and the timing of administration relative to the circadian phase (47, 48).

### 2.4 Circadian changes in night shift workers

Circadian adaptation poses greater challenges for night workers who remain influenced by external cues that encourage a daytime routine. Consequently, many individuals on night shifts exhibit different levels of circadian adaptation to their work hours. Complete adaptation, without intentional intervention, is rare in typical work environments, as shown by the inability of core body temperature, melatonin, and cortisol rhythms to align with a night schedule (32). According to studies, there are some differences in the components of circadian system when comparing night and daytime workers. For instance, cortisol levels are one of these differences. Weibel and colleagues has shown that (25). In spite of night workers, daytime workers have a minimal cortisol level early in the night and their maximal values around the regular time of awakening (25). Furthermore, in these workers melatonin is in its maximal levels at night during the middle of the sleep episode while its levels cannot even be detected during the day (25). Other investigations show that full adaptation of the circadian pacemaker to night shift work is likely to happen in only a small percentage (3%) of workers, even if they follow a consistent night shift schedule. Around 25% of workers might experience partial adaptation to night work, while the majority (approximately 72%) would not show any circadian adjustments. Nevertheless, the precise rate of circadian adaptation to night shift work is still under debate (26).

### 2.5 Night shifts and health issues

As mentioned before, night shifts cause health problems through interfering with the circadian rhythm and hormonal levels. In this regard, a diversity of studies has tried to explain the correlation of night shifts with diseases. Cardiovascular diseases are confirmed to occur as a consequence of prolonged night shifts and disrupted circadian system. Coronary heart disease (CHD) is one of the most important classes of these diseases which is approved to correlate with night shifts. A study on nurses shows that in women employed as registered nurses, an extended period of rotating night shift work was linked to a statistically significant, albeit minor, rise in the risk of CHD (49). A similar study also indicated that exposure to night shifts, both in the short term and over a lifetime, was linked to a higher risk of atrial fibrillation (AF), independent of genetic predispositions to AF. Moreover, working night shifts also raised the risk of coronary heart disease (CHD), but did not elevate the risk for stroke or heart failure (HF) (50). Yang et al. (51) also worked on myocardial infarction and found that working night shifts more frequently and for extended periods was linked to a greater risk of heart attacks and interestingly, longer rest days could not decrease myocardial infarction risk compared to those who rest 1 day (51). Studies on neurodegenerative diseases has also shown a strong relation. For instance, Chen and colleagues worked on women nurses and found out that in comparison to nurses who have never worked rotating night shifts, those who have worked night shifts for 15 years or longer exhibited a 50% reduced risk of developing Parkinson’s disease, even after controlling for age and smoking (52). They also presented that the amount of sleep was linked to the risk of Parkinson’s disease, with a relative risk of 1.84 found when comparing nurses who got 9 or more hours of sleep each night to those who slept 6 h or less. This information implies that working night shifts might offer some protection against Parkinson’s disease, or that difficulty with night shift work could indicate an early sign of the disease (52). Another study on 1,248 participants showed that the likelihood of developing dementia was greatest among those who worked night shifts consistently, followed by those with irregular shifts (53). Alzheimer’s disease events were documented in 474 participants throughout the follow-up period of this study and after applying final multivariate adjustments to the model, workers who consistently worked night shifts remained at the greatest risk (53).

In metabolic disease point of view, several study groups have explored the correlation. Lim et al. (28) are one of these groups which studies 494 Malaysian manufacturing workers, aged 40–65 years old. They found out that the occurrence rate of Metabolic Syndrome was found to be 37%. Working night shifts independently raised the risk of developing MetS by two times (28). Workers on the night shift also indicated notably worse sleep quality, extended time taken to fall asleep, reduced sleep duration, disturbances during sleep, and difficulties functioning during the day (28). Another study on 303 participants also showed that night shift workers exhibited higher levels of hs-CRP compared to those on day shifts (54). Additionally, there were significant increases in triglycerides (TG) and fasting blood sugar (FBS). About 6.5% of night shift employees had a waist circumference exceeding 40 inches. It was noted that night shift workers who had elevated hs-CRP also showed a significantly larger waist circumference and higher FBS levels. Moreover, 3.57% of the night shift workers met the three criteria for a diagnosis of metabolic syndrome (54). Another study on nurses also confirmed that the overall occurrence of MS was 9.0% (36 out of 402) for night-shift workers, compared to 1.8% (6 out of 336) for those who worked during the day. The yearly incidence rate of MS was 2.9% among night-shift employees, while it was 0.5% for daytime workers (55).

### 2.6 Night shifts and cancer

Unfortunately, a great body of evidence has confirmed that night shifts and its issues are able to cause a diversity of cancers. For example, Viswanathan et al. (56) worked on 515 women and concluded that women who work long-term rotating night shifts face a considerably higher risk of developing endometrial cancer, especially if they are overweight. They believe this heightened risk may be due to how melatonin affects hormonal and metabolic factors (56). Breast cancer is also another important cancer which its pathology can be related to night shifts. Davis and colleagues worked on 813 individuals and observed that the risk of breast cancer was elevated in individuals who often had trouble sleeping during the time at night when melatonin levels are usually at their peak (57). A study group (23) tried to find a correlation between colorectal cancer (CRC) risk and shift nights. They conducted a prospective study to investigate the link between working rotating night shifts and the risk of CRC in female participants of the Nurses’ Health Study. During the follow-up period from 1988 to 1998, they recorded 602 new cases of CRC among 78,586 women (23). In comparison to women who have never worked rotating night shifts, those who have worked them for 1 to 14 years or for 15 years or more have a multivariate relative risk of colorectal cancer of 1.00 (23). Finally they concluded that the information indicates that women who work a rotating night shift for at least three nights each month over a period of 15 years or more may face a heightened risk of developing colorectal cancer (23). In prostate cancer point of view, a meta-analysis was conducted in 2015 which included 2,459,845 participants from eight published studies. The examination of all studies indicated that working night shifts was linked to a notably higher risk of developing prostate cancer (58). A rise in night-shift work lasting five years was found to be significantly linked to a 2.8% higher risk of developing prostate cancer (58).

## 3 Methodology and scope of the review

This study employs a narrative review methodology to synthesize and critically examine the existing literature on nurse night shift work and the risk of gastrointestinal cancers. Unlike systematic reviews, which follow a rigid protocol for study selection and data extraction, narrative reviews offer a more flexible and interpretative approach, enabling a broader discussion of themes and concepts across diverse studies. We agree that conducting a systematic review for each new mechanism of action (MoA) is appropriate. While a systematic review could provide a highly focused analysis of individual MoAs linking night shift work to gastrointestinal cancer risk, our current approach as a narrative review aims to synthesize diverse mechanisms holistically. Given the multifactorial nature of this relation—melatonin suppression, encompassing circadian rhythm disruption, inflammatory pathways, and metabolic alterations—a narrative review allows us to integrate findings from various studies, highlight overarching patterns, and identify knowledge gaps. In addition, we recognize the value of systematic reviews focusing on specific MoAs and have emphasized in the revised manuscript that future systematic investigations could further explore distinct mechanisms in greater depth. The literature selection was guided by a structured search strategy, prioritizing nurse-specific studies. However, due to the limited availability of research exclusively focused on nurses, relevant studies from broader populations were also included to provide comparative context and a more comprehensive understanding of the subject matter. To ensure methodological rigor, we adhered to established best practices for conducting narrative reviews. Literature searches were conducted in PubMed, Scopus, and Web of Science, focusing on peer-reviewed epidemiological studies published in English. Predefined keywords and inclusion criteria were applied to identify relevant studies. The selected literature was then analyzed thematically to identify key patterns, challenges, and insights pertinent to nursing practice. This approach allows for a nuanced discussion of the topic while maintaining transparency in the selection and synthesis of evidence.

## 4 Correlation between night shift and risk factors of gastrointestinal cancers

### 4.1 Obesity

**Figure 2:**
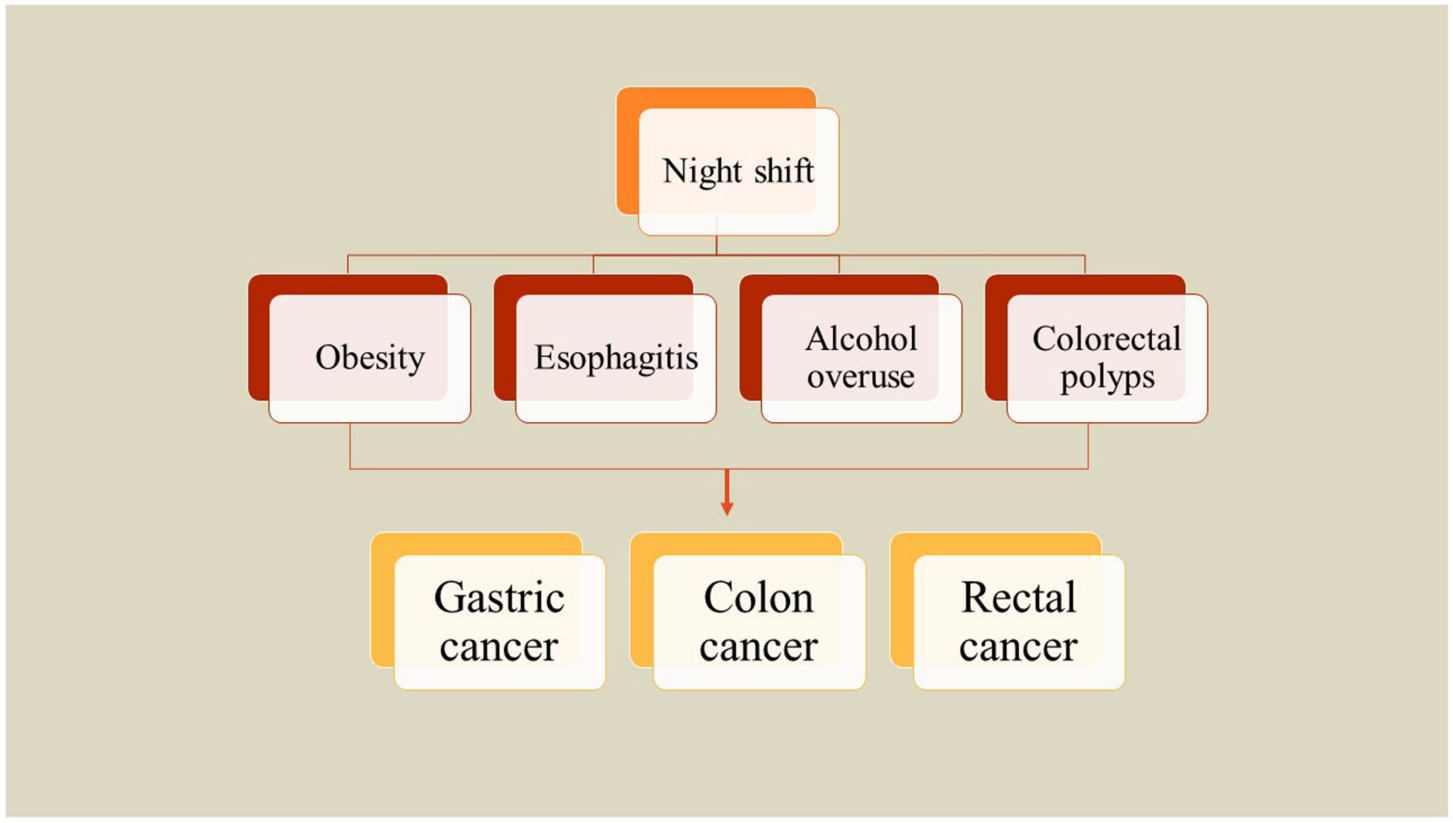
Correlation between night shift and risk factors of gastrointestinal cancers.

The incidence of obesity has surged significantly over the last thirty years, consequently increasing the burden of cancers linked to excessive body fat (27). A substantial amount of research indicates a correlation between obesity and heightened risks of developing gastrointestinal cancers. At present, three primary hypotheses are under examination to explain the association between excess body fat and the risk of gastrointestinal cancer which included changes in signaling pathways of insulin and IGF-1, obesity-mediated chronic low-grade inflammation, and modifications in the metabolism of sex hormones (27). Moreover, obesity may not only facilitate carcinogenesis but also influence the biological behavior of existing tumors. For example, individuals diagnosed with colon cancer and pancreatic cancer who are overweight or obese have been reported to experience worse survival outcomes (59, 60). To investigate the relationship between rotating night shift work and body mass index (BMI) as well as abdominal fat accumulation, Peplonska et al. (29) conducted a cross-sectional study involving 724 female nurses and midwives aged 40 to 60 years in Łódź, Poland, from 2008 to 2011. The study comprised 354 participants working rotating night shifts and 370 daytime workers. The findings indicated that cumulative night shift work is significantly associated with BMI, waist circumference (WC), hip circumference (HC), and waist-to-height ratio (WHtR). Specifically, BMI was observed to increase by 0.477 kg/m^2^ for every 1,000-night shifts worked and by 0.432 kg/m^2^ for every 10,000 h of night work. Additionally, WC and HC increased by 1.089 cm and 0.72 cm, respectively, for the same metrics, while WHtR increased by 0.007 cm for both. Notably, both current and cumulative night shift work were linked to a higher prevalence of obesity (defined as BMI ≥ 30 kg/m^2^), with an odds ratio of 3.9 (95% confidence interval: 1.5–9.9) observed in women who reported working eight or more night shifts per month (29). A similar investigation on a total of 3,871 workers from five companies has assessed the correlation between obesity indices and different types of night shifts in China. Findings demonstrated that night shift employees have higher chances for overweight and obesity, with odds ratios (ORs) of 1.17 and 1.27, respectively. Furthermore, a notable yet marginal association exists between abdominal obesity and night shift work, indicated by an OR of 1.20. A positive correlation was observed between the duration of night shift employment and the prevalence of overweight or abdominal obesity. Specifically, those engaged in permanent night shift work faced the greatest likelihood of being classified as overweight who exhibit increased abdominal obesity. Irregular night shift schedules also demonstrated a significant relationship with overweight status. However, the correlation with abdominal obesity was marginal. Conversely, rotating night shift work did not show a significant association with these health parameters. Consequently, both permanent and irregular night shift work are more strongly linked to overweight and abdominal obesity compared to rotating night shifts (61). Brum et al. (62) also investigated the relationship between obesity, quality of life, and shift work among healthcare staff in Brazil. They reported that those who worked night shift were older than those who worked day shift and had higher income. While night shift workers had lower sleep hours, they showed increased body mass index, weight, and abdominal circumference compared to the day shift workers. Night shift employees exhibited nearly three times the likelihood of experiencing abdominal obesity compared to their day shift counterparts, irrespective of age and gender. Analyses of the MCTQ indicated that night shift workers had reduced sleep duration both on workdays and off days, which was linked to a heightened level of social jetlag. Furthermore, social jetlag was found to be associated with obesity. However, no significant differences in quality of life were observed between the two shifts. Consequently, it can be concluded that night work serves as a risk factor for abdominal obesity, with night shift workers experiencing greater social jetlag that correlates with the incidence of obesity (62) (Figure 2).

### 4.2 Alcohol consumption

Since 1988, the International Agency for Research on Cancer (IARC) has classified alcohol as a Group I carcinogen (63, 64). According to a report by the World Health Organization (WHO) released in 2018, approximately 3 million fatalities were attributed to alcohol consumption in 2016, accounting for 5% of total deaths, with around 13% of these deaths linked to cancer (65). Ethanol, the primary ingredient in alcoholic beverages, is metabolized into acetaldehyde upon consumption, which is subsequently oxidized into acetate (66). Animal studies have shown that both ethanol and acetaldehyde have been implicated in carcinogenesis. Acetaldehyde is particularly harmful as it causes irreversible damage to DNA, inhibits DNA repair processes, and reduces telomere length (67). Additionally, alcohol consumption can lead to the production of reactive oxygen species (ROS) and impair the functionality of scavenger systems, resulting in oxidative stress that contributes to genetic instability (68). Furthermore, alcohol’s carcinogenic properties may also arise indirectly through its association with nutritional deficiencies that compromise the integrity of DNA (69). A research study was conducted to assess the prevalence of night-shift employment and its correlation with alcohol use disorders (AUDs) and health-related quality of life (HRQL) among adult workers in Korea. This analysis that involved 26,895 participants aged between 20 and 59 years indicated a significant interaction between gender and employment status concerning AUDs, revealing that women exhibit greater vulnerability to the impacts of night work compared to men, although this trend was not observed in relation to HRQL. Specifically, female workers on night shifts presented a higher risk of developing AUDs compared to their daytime counterparts, while no similar effect was identified among male night workers. Additionally, night workers demonstrated a lower HRQL with respect to depression when compared to those working daytime shifts, whereas those with regular day-night shifts appeared to be shielded from depressive symptoms. Therefore, it is highlighted that while female night workers face an increased risk of AUDs and diminished HRQL, male night workers do not share these vulnerabilities, reinforcing the notion that women are more susceptible to the adverse effects of night work (70).

### 4.3 Esophagitis

A cross-sectional analysis involving 6,040 male shipyard workers was conducted, during which Esophagogastroduodenoscopy examinations were carried out to assess the odds ratios of erosive esophagitis in relation to their status of night-shift work. The results indicated an increased prevalence of erosive esophagitis among night-shift workers. Multiple logistic regression analyses identified night-shift work, obesity, smoking, and alcohol intake of 140 grams or more per week as significant risk factors for the condition. Conversely, an infection with *Helicobacter pylori* was found to have a negative correlation with erosive esophagitis. Thus, it can be concluded that night-shift work is a potential risk factor for erosive esophagitis (71). Another research investigation was conducted to examine the relationship between shift work and the occurrence of reflux esophagitis. This cohort study involved 140,553 participants who were monitored at least once between the years 2012 and 2018. Over the course of 469,217.2 person-years of follow-up, 35,185 participants were identified as having developed new cases of reflux esophagitis. The multivariable adjusted hazard ratio for these incident cases indicated a value of 1.09 (with a 95% confidence interval of 1.04–1.13) when comparing individuals engaged in shift work to those working fixed daytime hours. Younger individuals who aged 18 to 39 years and females are shown to express a notably stronger association. In summary, the findings suggest a significant correlation between shift work and the incidence of reflux esophagitis, with pronounced effects evident particularly in younger and female populations (72). In another cross-sectional study, participants underwent esophagogastroduodenoscopy from January 2011 to December 2018 to explore the correlation between shift work and reflux esophagitis. The findings indicated that out of 247,450 participants, 20.1% were diagnosed with reflux esophagitis. The fully adjusted multivariate odds ratio for overall reflux esophagitis in individuals engaged in shift work, relative to those with fixed daytime employment, was calculated at 1.15. The odds ratios for Los Angeles Classification A (LA-A) for regular day and night shifts, as well as irregular shifts, compared to fixed daytime schedules were 1.14 and 1.26, respectively. Notably, no statistically significant relationship has been found between any shift work pattern and a severity classification of ≥LA-B. These results suggest a potential link between shift work, particularly rotating and irregular shifts, and mild reflux esophagitis (≤LA-A) when contrasted with fixed daytime work schedules (73). Lee and colleagues conducted a study involving 964 non-shift workers and 290 shift workers aged between 22 and 40 years at an electronics company to examine the relationship between gastritis and shift work. Their findings indicated that night shift workers reported higher instances of indigestion compared to their non-shift working counterparts, alongside a greater prevalence of gastritis. The likelihood of developing gastritis was notably increased among shift workers, with an odds ratio (OR) of 2.24 when compared to non-shift workers. However, among the seven identified subtypes of gastritis, only superficial gastritis demonstrated a significant correlation with shift work. These results suggest that shift workers not only experience a higher incidence of gastritis but also report more gastrointestinal symptoms, particularly indigestion, thus indicating that shift work adversely impacts the gastrointestinal system (74).

### 4.4 Colorectal polyps

Um et al. conducted a study to investigate the impact of night shift work on the occurrence of colorectal polyps, which have the potential to develop into colorectal cancer. The research involved a sample of 299 men aged between 40 and 60 years, sourced from two university hospitals. The study assessed various factors, including participants’ lifestyles, work histories, work patterns, and colonoscopy findings. The findings revealed that the prevalence of colorectal polyps among night shift workers was 53.0%, compared to 33.5% among those who did not work night shifts. After controlling for variables such as age, smoking habits, dietary practices, family history of colorectal cancer, obesity, and job type, night shift emerged as a significant risk factor for colorectal polyps. Consequently, the study concluded that the likelihood of developing colorectal polyps is higher in individuals who engage in night shift work, with an increased risk observed in older age groups (75).

## 5 Night shift work and risk of gastrointestinal cancers

### 5.1 Gastric cancer

A case–control study involving 374 newly diagnosed stomach adenocarcinoma cases and 2,481 population controls was conducted to investigate the relationship between night shift work and the risk of stomach cancer. The findings indicated that 25.7% of the cases and 22.5% of the controls reported a history of working night shifts. The analysis revealed a weak, non-significant positive correlation between having worked at least one year in permanent night shifts and the incidence of stomach cancer when compared to individuals who had never worked night shifts. Notably, a non-linear ‘U’ shaped relationship was observed regarding the cumulative duration of permanent night shifts, with the highest cancer risk noted in the intermediate duration group. In contrast, no significant association was found with those who had worked rotating night shifts, nor was there any discernible trend based on the cumulative duration of such shifts. Consequently, the study concludes that there is insufficient evidence to establish a definitive link between night shift work and the risk of stomach cancer (76). The MCC-Spain study reported an association between extreme sleep durations and gastric cancer risk (77). However, a key concern in interpreting these findings is the potential for reverse causality—where undiagnosed tumors could contribute to sleep disturbances such as insomnia or hypersomnia. Ideally, studies should exclude cancer cases diagnosed within the first 1–2 years of follow-up to mitigate this bias. If such exclusions were not applied, there is a possibility that early tumor-related symptoms influenced sleep patterns rather than sleep duration being a causal factor in gastric carcinogenesis. Future studies should incorporate sensitivity analyses excluding early-diagnosed cases to strengthen the evidence for a causal relationship between sleep duration and cancer risk (Table 1).

**Table 1:** Studies investigating the role of night shift work on the risk of gastrointestinal cancers.

Cancer type	Type of shift	Subjects / number	Findings	Ref.
Stomach adenocarcinoma	Permanent and rotating night shift	374 cases and 2,481 controls from the MCC-Spain study	No significant association has been found between night shift work and stomach cancer risk	Gyarmati et al. (76)
Colorectal cancer	Rotating night shift	1,397 cases from the Nurses’ Health Study	Rotating night shift may increase colorectal cancer risk in women with abnormal insulin receptor pathways	Shi et al. (24)
Colorectal cancer	Rotating and permanent shift work	1,626 cases and 3,378 controls from the MCC-Spain study	Rotating shift work may increase the risk of colorectal cancer, especially after long-term exposures	Papantoniou et al. (78)
Rectal cancer	Rotating night shift work	190,810 women and 1,965 incident of colorectal cancer cases from the Nurses’ Health Study (NHS) and NHS2	Risk of rectal cancer significantly increased with shift work duration	Papantoniou et al. (31)
Colorectal adenoma	Rotating night shift work	56,275 cancer-free participants of the Nurses’ Health Study II	No association has been found between rotating night shift work or sleep duration and risk of colorectal adenoma in women	Devore et al. (79)
Colorectal cancer	Night shift work	6,903 participants from the Heinz Nixdorf Recall Study (HNR) and the Study of Health in Pomerania (SHIP)	Night-shift work was not associated with colorectal cancer, although the risk was increased for rotating shift work including nights in SHIP	Wichert et al. (80)
Colorectal cancer	Graveyard shiftwork	350 cases and 410 controls from Graveyard shift workers of an Australian population	No increase has been observed in risk of colorectal cancer among females who had worked in occupations involving shiftwork	Walasa et al. (81)
Colorectal cancer	Rotating shift work	8,199 male and female nurses and midwives from Australia, New Zealand and the United Kingdom	No association has been found between rotating shift work and colorectal cancer in nurses and midwives	Wickremaratne et al. (30)

### 5.2 Colon cancer

A study involving 77,470 women engaged in night work identified 1,397 cases of colorectal cancer. Data on IRS1 and IRS2 was accessible for 304 and 308 cases, respectively. To explore the relationship between the expression of insulin receptor substrates and the risk of colorectal cancer among night-shift workers, immunohistochemistry (IHC) was used to assess tumor expression levels of IRS1 and IRS2. The findings indicated that women who worked night shifts for 15 years or more exhibited a slight trend toward an increased overall risk of colorectal cancer compared to those who never worked night shifts. Additionally, a longer duration of night work correlated with a heightened risk of IRS2-positive tumors, while no such association was found for IRS2-negative tumors. This suggests that IRS may play a significant role in the carcinogenic processes associated with night-shift employment (24). A study conducted by Papantoniou et al. (78) investigated the relationship between shift work history and the risk of colorectal cancer (CRC) in a case–control study in Spain. The research involved 1,626 new CRC cases alongside 3,378 randomly selected control participants across 11 regions of Spain. The findings indicated that individuals who had engaged in rotating shift work (morning, evening, and/or night shifts) faced a higher risk of developing CRC compared to those who only worked daytime hours. Conversely, those who had worked permanent night shifts (three or more nights a month) did not show an increased risk for CRC. The odds ratio grew with the cumulative duration of rotating shift work over a lifetime and was found to be highest among individuals in the upper quartiles of exposure. This suggests that long-term exposure to rotating shift work may significantly elevate CRC risk (78).

A study examined the relationship between rotating night shift work and the risk of colorectal cancer in two prospective female cohorts: the Nurses’ Health Study (NHS) and NHS2, with a follow-up period of 24 years. The analysis included a total of 190,810 women, during which 1,965 new cases of colorectal cancer were documented. The findings indicated no significant correlation between the duration of rotating night shifts and the risk of colorectal cancer within these cohorts. However, further analysis within the NHS revealed an increased risk of rectal cancer associated with long-term rotating night shift in those who worked this way for 15 years or more. Altogether, the study found no conclusive evidence linking rotating night shift work to an increased risk of colorectal cancer in these extensive cohorts of nurses. Nonetheless, the observed increase in rectal cancer risk with extended shift work duration suggests that prolonged circadian disruption may contribute to the development of rectal cancer (31). In a similar investigation, Devore and colleagues have investigated the correlation between sleep duration and night shift and colorectal adenoma in 56,275 cancer-free participants of the Nurses’ Health Study II. These individuals had their first colonoscopy or sigmoidoscopy between 1991 and 2011. The analysis revealed no correlation between the length of rotating night shift work and the incidence of colorectal adenoma. Specifically, women who engaged in rotating night shift work for an extended period (10 years or more) exhibited a risk of adenoma comparable to that of women with no prior experience in such work. Additionally, findings indicated that there were no significant associations between either short or long sleep durations and the risk of developing adenomas. This trend persisted when the relationships were assessed based on the anatomical location and subtype of the adenomas. Consequently, it is found that there is no link between rotating night shift work or sleep duration and the risk of colorectal adenoma in women (79).

Wichert et al. (80) conducted research on the relationships between exposure to shift or night work and the incidence of colorectal cancer (CRC) using two population-based cohort studies conducted in Germany: the Heinz Nixdorf Recall Study (HNR) and the Study of Health in Pomerania (SHIP). Their analysis included up to 6,903 participants and examined the cohorts both collectively and separately. The results from the pooled analysis indicated that there was no heightened risk of CRC among men engaged in night shifts. However, within the male participants of the HNR, an increased risk estimate for distal colon cancer was identified among shift workers, including those who did not work night shifts, whereas night workers did not show an elevated risk. Conversely, the SHIP data revealed higher risk estimates for colorectal cancer associated with rotating shift work that included night shifts, as well as for prolonged exposure among men. Thus, it is implied that while night shift work was not linked to CRC, the findings from SHIP suggested a potential increase in risk related to rotating shift work that encompasses night shifts (80). Another investigation has explored the relationship between two forms of shift work, graveyard shifts and early-morning shifts, and six mechanistic variables related to shift work, such as nocturnal light exposure and circadian phase shifts, in relation to the risk of colorectal cancer among women. This study, which was based on a case–control design involving 350 cases and 410 controls within an Australian population, revealed that prolonged exposure (>7.5 years) to graveyard shift work was not linked to an elevated risk of colorectal cancer. Furthermore, no significant associations were found for any of the other seven shift work variables assessed. Consequently, the study concludes that there is no evidence to suggest an increased risk of colorectal cancer among women employed in shift work roles (81).

Simons-Linares and colleagues conducted an observational study involving patients who underwent screening colonoscopy at a hospital from September 2015 to January 2016, focusing on a comparison between night-shift workers and non-night-shift workers. The sample included 314 patients with a mean age of 58 years, of whom 53% were male. Among these patients, 35% (110 individuals) were identified as night-shift workers. The incidence of colon cancer was recorded at 0.6%, with two cases of carcinoma identified. The overall polyp detection rate was 43%, while the adenoma detection rate stood at 33%. Upon comparing individuals who had never worked night shifts with those who had worked night shifts for varying durations (1–14 years and over 15 years), no significant increase in the risk of colonic adenomas was observed. Furthermore, no notable risk for colonic adenomas was found across the four groups of night-shift workers categorized by years of night work (1–5 years, 5.1–10 years, 10.1–15 years, and over 15 years). Notably, after adjusting for night-shift work, the use of aspirin was determined to be protective against colon adenomas specifically for night-shift workers, without any significant effect on non-night-shift workers. The study concluded that employment in night-shift work does not elevate the risk of developing colonic adenomas or colorectal cancer (30).

The HUNT study identified a male-specific increased risk of right colon cancer (HR = 1.93) associated with night shift work (82). However, this analysis did not account for potential sex-dependent confounders such as occupational exposures common in male-dominated industries (e.g., chemical or carcinogen exposure) or behavioral differences, including smoking and alcohol consumption patterns. These unmeasured factors could contribute to the observed sex differences. Future research should incorporate stratified analyses based on occupational type and lifestyle factors to determine whether the association between night shift work and colon cancer risk remains independent of these confounders. Adjusting for these variables in future cohort studies would help clarify whether the observed male-specific risk is attributable to night shift work itself or other environmental and behavioral exposures.

The Nurses’ Health Studies reported null associations between night shift work and overall colorectal cancer (CRC) risk (23, 77). One potential explanation is the healthy worker effect, where nurses, as healthcare professionals, may engage in healthier behaviors such as better diet, lower smoking rates, and increased physical activity, which could mitigate cancer risk. Additionally, nurses may have higher adherence to CRC screening guidelines, leading to earlier detection and possibly lower incidence rates. Comparisons between nurses and other professional groups, such as manufacturing or transportation workers, who may have different occupational exposures and lifestyle factors, could help clarify the extent of this bias. While some studies on non-healthcare shift workers have reported positive associations between night shift work and CRC, direct comparisons across professions remain limited. Future research should explore these differences by analyzing CRC risk estimates across diverse occupational groups while adjusting for screening behaviors and lifestyle factors.

## 6 Conclusion

Circadian rhythm is a sophisticated system which enables organisms to adapt to their external environment and use their energy in the most cost-effective way. Circadian rhythm is considered as an important basis of a living organism because of its pivotal role in affecting aging, metabolism, and other processes. Due to the importance of this system, it’s essential to study the factors which are able to cause a disturbance in it and interfere with many cellular and molecular mechanisms.

In humans, one of the most common factors that affect the circadian rhythm, is night shifts and the lack of sleep during the night and thus, we herein reviewed studies on the role of night shift work on the risk of gastrointestinal cancers. What we have concluded during reviewing this evidence is described below:

1. In gastric cancer point of view, there is only one study investigating this correlation which has not find any strong relation and thus, further research are required in gastric cancer field.
2. In colon cancer point of view, studies have shown that there is a higher risk of CRC (specially IRS2-positive tumors) in men and women who has worked night shifts (for 15 years or more).

Melatonin suppression has been proposed as a key mechanism linking night shift work to gastrointestinal carcinogenesis. Observational studies, such as Garcia-Saenz et al., have suggested an association between blue light exposure and colorectal cancer risk, presumably due to its impact on melatonin levels (83). However, the current body of evidence is primarily based on epidemiological data, and direct experimental studies assessing causality remain limited. While animal models have demonstrated the tumor-suppressive role of melatonin, human experimental data are lacking. Future research should incorporate controlled experimental studies to evaluate the direct impact of melatonin suppression on gastrointestinal carcinogenesis, potentially through interventional trials assessing melatonin supplementation as a protective factor in night shift workers. A key limitation of this review is the variability in how night shift work is defined across different studies, potentially leading to exposure misclassification. While some studies distinguish between rotating and fixed night shifts, others group all shift types together, making it difficult to draw definitive conclusions about cancer risk. For instance, the HUNT study (82) reported a significant association between long-term night shift work and right colon cancer in men, whereas the German cohort (80) found no significant association. These discrepancies highlight the need for future studies to use standardized definitions of night shift work and conduct sensitivity analyses that focus specifically on long-term fixed night shift workers to better understand the underlying risks. Future research should also explore whether the duration and intensity of shift work further influence gastrointestinal cancer risk. According to these findings, there is still a long way till we can come to a definite conclusion about the relation between night shifts and the risk of gastrointestinal cancers. More research should be conducted on other GI cancers including pancreatic, oral, and esophageal cancers. Furthermore, in the discussed cancers, further research can reveal the molecular basis of this correlation and open new windows to overcoming the disadvantages of night shifts.
